# Study protocol of an RCT of EMOTION: An indicated intervention for children with symptoms of anxiety and depression

**DOI:** 10.1186/s40359-016-0155-y

**Published:** 2016-09-26

**Authors:** Joshua Patras, Kristin Dagmar Martinsen, Solveig Holen, Anne Mari Sund, Frode Adolfsen, Lene-Mari Potulski Rasmussen, Simon-Peter Neumer

**Affiliations:** 1 The Regional Centre for Child and Youth Mental Health and Child Welfare – Northern Norway, RKBU Nord UiT Norges arktiske universitet, 9037 Tromsø, Norway; 2 The Center for Child and Adolescent Mental Health – Eastern and Southern Norway, Postboks 4623 Nydalen, 0405 Oslo, Norway; 3 The Regional Centre for Child and Youth Mental Health and Child Welfare – Central Norway, Pb 8905, MTFS, N-7491 Trondheim, Norway; 4 St. Olavs Hospital, Trondheim University Hospital, Prinsesse Kristinas gate 3, 7030 Trondheim, Norway

**Keywords:** Indicated prevention, Anxiety, Depression, Internalizing, RCT, Children, Effectiveness, Implementation, Cognitive behavioral therapy, Schools

## Abstract

**Background:**

High levels of anxiety and depression are common psychological symptoms among children and adolescents. These symptoms affect young people in multiple life domains and are possible precursors of long-term psychological distress. Despite relatively high prevalence, few children with emotional problems are referred for clinical treatment, indicating the need for systematic prevention. The primary aim of this study is to evaluate an indicated preventive intervention, EMOTION Coping Kids Managing Anxiety and Depression (EMOTION), to reduce high levels of anxiety and depressive symptoms.

**Methods/Design:**

This is a clustered randomized controlled trial involving 36 schools, which are assigned to one of two conditions: (a) group cognitive behavioral intervention EMOTION or (b) treatment as usual (TAU). Assessments will be undertaken at pre-, mid - intervention, post-, and one year after intervention. The children (8–11 years old) complete self-report questionnaires. Parents and teachers report on children. The primary outcome will be changes in depressive and anxiety symptoms as measured by the Short Mood and Feelings Questionnaire (SMFQ) and Multidimensional Anxiety Scale for Children (MASC) respectively. Secondary outcomes will be changes in self-esteem, quality of life, and school and daily functioning. Observers will assess implementation quality with ratings of fidelity based on video recordings of group leaders leading the EMOTION group sessions.

**Discussion:**

The present study is an important contribution to the field regarding working with children with symptoms of anxiety and depression. The results of this study will provide an indication whether or not the EMOTION program is an effective intervention for the prevention of later depression and/or anxiety in children. The study will also provide information about the EMOTION program’s effect on quality of life, self-esteem, and school functioning of the children participating in the study. Finally, the project will provide insight into implementation of an indicated intervention for school-aged children within Norwegian health, education, and mental health services.

**Trial registration:**

Clinical Trials NCT02340637, Registered on June 12, 2014, last updated on January 15, 2015. Retrospectively registered.

## Background

 Evidence-based approaches have typically been disorder-specific targeting one disorder at a time, for example, the Coping Cat program for anxious youth [1] and the Taking ACTION program for depressed youth [2]. Anxiety and depression, however, may share a common predisposition that, in the presence of stress, lead to the expression of anxiety, depression, or both [3]. Developing integrated programs that target multiple but related problems, a transdiagnostic approach, has great appeal and will make evidence based interventions more available to children in need. While comparable interventions have been developed in the US [4, 5], there is to our knowledge no such intervention available for this age group in Norway today. Following international guidelines for the initial management of depression in primary care [6] and in close collaboration with the program developers, the essential core elements of the Coping Cat program and Taking ACTION program have been combined. The resulting program, EMOTION Coping Kids Managing Anxiety and Depression (EMOTION) [7], is an intensive course of 20 sessions that run twice per week for 10 weeks; this is explained in detail in the Intervention section of this manuscript. In addition to the 20 sessions for the children, the parents participate in seven meetings to learn strategies to support their children; the children also attend four of the parent’s meetings.

### Indicative intervention in a school setting

The EMOTION program is an indicated intervention. One challenge for indicated interventions is the need for a screening procedure to recruit youth to the intervention. Stigma associated with being selected due to indication is a potential problem, but in a pilot study of the EMOTION program described below [8], the children reported low experience of stigma and high user satisfaction; this result is similar to a systematic study of stigma for adolescents with depression [9]. Delivering interventions in schools will provide better access to children who might not otherwise receive services, as well as allowing for better collaboration between clinicians and school personnel, thus creating better continuity of care [6, 10]. Good attendance is another advantage of delivering the program in school [11], and the EMOTION pilot study reflected this with very low attrition rates. Delivering interventions in schools has advantages across a range of emotional and behavioral problems [12, 13].

### The pilot study of EMOTION Coping Kids

The EMOTION pilot study took place in one elementary school in rural Norway. The pilot study investigated recruitment strategies, appropriate screening instruments, attendance rates, social stigma, and user satisfaction with the EMOTION program [8]. Twenty-two children nominated themselves to join the intervention and eleven of them who had elevated symptoms of anxiety, depression, or both (≥0.50–2.00 *SD*) joined the EMOTION groups. There were no dropouts from the EMOTION groups and overall participation was 98 % for the children and 75 % for the parents. User satisfaction was high and stigma was low. Feedback from the children, parents, and group-leaders was used to revise the content of the intervention.

Based on the pilot study, the EMOTION intervention has the potential to be an effective and practical transdiagnostic indicative intervention. Results will depend on delivering the intervention with high fidelity in a school setting. Such an intervention will be innovative and valuable from a public health perspective as it may prevent the development of very common and disabling mental health problems for a large number of youth in need.

## Method/Design

The present study is a clustered, two-armed RCT delivered in schools from three regions in Norway: southern, central, and northern. The stages of the enrollment, intervention, and assessment can be seen in Table 1.

**Table 1:**
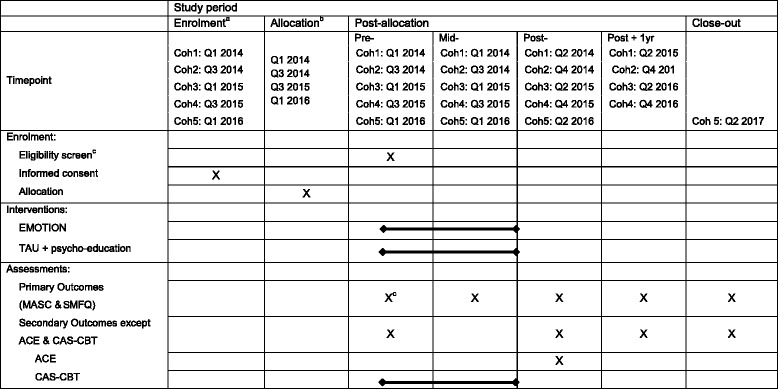
SPIRIT table for evaluation of the EMOTION Coping Kids RCT

^a^Enrollment occurs in the semester prior to delivery of the intervention. Each cohort represents a group of children recruited during the semester

^b^Allocation (randomization) is conducted at the school-level, therefore allocation reflects when new schools joined the study

^c^Study eligibility for individual children is based on their scores on the MASC and SMFQ (primary outcome measures)

### Eligibility criteria

School-aged children between eight and eleven years old from 3^rd^, 4^th^, 5^th^, and 6^th^ grades are eligible for participation. School eligibility is determined by region of the country and municipality. The primary study sites are in the northern, central, and southeastern regions of Norway. In northern Norway, where the population is less dense, two municipalities (i.e., Bodø and Tromsø) are locations for school recruitment. In the central and southeastern sites respectively, municipalities in closer proximity to one another are large enough to provide enough participating schools.

The recruitment period is lasting for two years, starting in spring 2014 and is going until summer 2016. School-size is a recruitment criterion. The participating schools need to have at least one full class in each grade to increase the probability of recruiting enough children to run an EMOTION group. The intervention is offered once in the autumn and once in the spring term of each school year. The plan is to include an average of six children from each grade in each school every semester; a minimum of three children and a maximum of seven children per group will be allowed. The children are screened before, during, and after the intervention. In addition, participating children are measured one year after intervention to identify possible long-term effects. When the study is finished, the communities are free to implement EMOTION at the control schools. Children at the participating schools are recruited via an open invitation letter that is sent home to the parents; children are invited to participate if they and/or their parents consider themselves to have more sad or anxious feelings than their peers.

#### Inclusion criteria

The children who agree to take part in the study fill out the Short Mood and Feeling Questionnaire for depression (SMFQ) [14, 15] and Multidimensional Anxiety Scale for Children (MASC) [16]. All of the children who fill out the screening questionnaires who score at least one standard deviation above the expected mean score for either depression (SMFQ; *M* = 3.8, *SD* 3.6), anxiety (MASC; girls *M* = 46, *SD* 15; boys *M* = 39, *SD* 15), or both, are then invited to join the study.

#### Exclusion criteria

Children who may not benefit from a group-process intervention (e.g. severe cognitive or developmental challenges) are considered individually and the reasons for exclusion are documented according to Consort guidelines [17].

### Intervention care and comparison

#### Intervention

EMOTION is a group-based intervention to reduce the symptoms of anxiety and depression in school-aged children. Professionals who work in community health, special educational service, mental health care, or the schools deliver the intervention to children in 20 group sessions over 10 weeks. The professionals are given a three-day training in EMOTION and are referred to collectively as group leaders. The EMOTION intervention focuses on building skills and on anxiety or depression-related activities for the first 10 sessions. For depression, the first 10 sessions focus on psychoeducation, emotion regulation, and behavioral activation; a strategy to encourage behaviors that lead to fun and positive experiences. The latter 10 sessions focus on maintaining activation and cognitive restructuring. The anxiety-related activities for the first 10 sessions focus on building a fear hierarchy, while graduated exposure to fear-inducing situations are introduced in the latter 10 sessions. In order to support the children, parents are asked to attend seven parenting sessions. The children also attend half of the parenting sessions. The parenting sessions focus on creating a supportive home environment for the children as well as practicing new skills together with their children in the joint group meetings. A complete overview of the EMOTION program and its contents can be found in the published results from the pilot study [8].

In addition, the teachers and school-nurses in the schools are offered a half-day psycho-educative training aimed at providing a better understanding of children with anxiety and depression symptoms, as well as recommendations in how to support these children. In order to support the group leaders in the their delivery of the intervention they will receive regular supervision from a trained cognitive behavioral therapist. The supervisors will in-turn receive support from the study coordinating office (RBUP East and South). Group leaders are also asked to deliver video recordings of 20 % of their sessions to be scored for quality assurance.

#### Control

The teachers and school-nurses in the control schools are offered a half-day psycho-educative training that is equivalent to the training received by teachers and nurses in the intervention schools. Parents of children who score positive to a suicide screening question, are contacted by the research group. If the parents are worried about their child, they are encouraged to seek help from their regular GP or the school health nurse.

### Procedure

The study is registered at clinical trials (NCT02340637) and is funded by a competing grant from the Norwegian Research Council (NFR 228846/CR). Additional funding for the project is provided by The Center for Child and Adolescent Mental Health – Eastern and Southern Norway (RBUP East and South) and The Regional Centres for Child and Youth Mental Health and Child Welfare – Northern Norway and Central Norway (RKBU North and RKBU Central, respectively).

### Randomization

Participants are randomized at the school-level. Because schools are randomized only once when they join the study, study participants may know prior to offering their consent for participation whether they would receive EMOTION or be in a control school being offered treatment as usual (TAU). The schools will be matched prior to randomization based on geographic location (municipality) and school size. Randomization is to be conducted by coin flip and the results corroborated by an external expert cooperating with a member of the research support team at RBUP. Thirty-six schools are included to have a balanced design with an equal number of schools in each design arm and to ensure that the sample of students in the experimental condition is large enough to detect the expected effect size for the main outcome. Group sizes were limited to maximum seven students per group so that group leaders had a reasonable group size to manage. When the number of students who meet inclusion criteria exceeds the group maximum size, a group of seven students is randomly selected to fill the group.

### Blinding

Because of the nature of the current interventions, it is not possible for the participants to be unaware of the intervention condition they are assigned to after baseline. Furthermore, schools are randomized to either EMOTION or TAU prior to participation (see Randomization), so most participants are aware of the treatment condition prior to the baseline assessment.

### Outcomes

Data are collected at four time points: T1 (pre intervention), T2 (mid intervention), T3 (post intervention) and T4 (1-year post intervention follow-up). Participants from the intervention schools (children, parents, teachers, and group leaders) are sent links to the questionnaire via SMS, email, or in the case of the children, are given unique, confidential identifiers that they use to log in using school computer labs. Because of the children’s ages, an adult is present while they fill out the questionnaire in order to clarify potentially confusing questions. At least one parent of each participating child is asked to fill out the parent questionnaires, although both parents are encouraged to participate. Teachers also fill out a questionnaire about participating children in their classroom.

#### Primary outcomes

The primary outcomes of interest for this study are the changes in depression and anxiety from pre- to post-intervention and from pre-intervention to follow-up. The null hypotheses for the primary outcomes of this study is that there will be no significant differences in changes of depression or anxiety scores between the intervention and the control groups. Depression is assessed using the SMFQ [14], a 13-item measure assessing cognitive, affective, and behavioral-related symptoms of depression in children 8 to 18 years. Anxiety is assessed using the MASC [16], a 39-items self-report measure for adolescents between 8 and 19 years.

#### Secondary outcomes

Quality of life will be assessed using *Children Quality of Life Questionnaire* [Kinder Lebensqualität Fragebogen] (KINDL) [18] by both children and parents. It consists of 24 items and measures physical and emotional wellbeing, self-esteem, and social functioning.

The *Beck Youth Inventory* (BYI-II) [19] will measure the children’s sense of self, a 20-item sub-scale regarding the youth’s self-concept.

*The Emotion Regulation Checklist* (ERC) [20], is a 24-item parent-report measure of children’s self-regulation, which focuses on concepts of affective liability, intensity, valence, flexibility, and situational appropriateness. The checklist includes both positively and negatively weighted items rated on a 4-point Likert Scale.

*Brief problem monitoring* –teacher (BPM-T) and parent (BPM-P) form [21], are an 18-item and 19-item version respectively of the Child Behavior Checklist scale (CBCL). The BPM-T and -P provide a uniform problem scale assessing both behavioral and emotional problems in school.

*ACE Stigma and evaluation* sheet [22], 17-item questionnaire related to embarrassment about participating in the study and participant satisfaction with the program.

*Hopkins Symptoms Checklist* (HSCL-10) [23] is used to assess possible psychopathology among participating children’s parents.

*Competence and Adherence Scale for Cognitive Behavioral Therapy* (CAS-CBT) [24]. To measure treatment adherence and competence (fidelity) in the EMOTION intervention.

### Background questions

Demographic information is collected about the parent’s socio-economic status, educational level, recent negative life events in the family, and the child's somatic health. Background information is also gathered about the group leader’s education and work experience related to group-based interventions for children.

### Recruitment and participation

Recruitment and participation data will be reported for available data from baseline.

### Participant retention

Contact with participants families is maintained by school personnel and reminders to fill out questionnaires are sent via email or sms.

### Data management

Data are collected and managed by an independent data collection team at the primary sponsor site. Data analysis and cleaning will be performed by study investigators. Data will be stored on a secure server during the study and analysis of results. Project staff will have access to the final trial dataset. Following the study, the data will be anonymized and archived according to Norwegian law.

### Data analysis

The study is carried out in schools and therefore data analyses will be conducted in a multilevel modeling framework to account for non-independence of the participants at the school level.

#### Sample size

The power estimation was based on an equation recommended accounting for a multi-level approach [25]. A total of 559 children (see Table 2) were deemed necessary to test the effectiveness of the intervention given the desired significance level (0.05), required power (0.80), and the following conditions:

**Table 2:** Number of participants and clusters required in a multilevel study

Estimated ICC	Calculated DEFF	Number of children	Number of schools
0.00 - Base model	1	260	15
0.05 - Two-level model	2.15	559	23(36)^a^

*ICC* the intraclass correlation coefficient, *n* number of pupils, *DEFF* design effect = 1 + (nc - l)*ICC, (*nc* average number of individuals in a school = 24); The first value (260) is from Altman [26], page 456, and the next figures are multiplied with the calculated DEFF value

^a^The first number denotes the required sample size given the power calculation. The parenthetical number denotes the actual number of schools recruited for the study based on other practical considerations

- anticipated effect size in anxiety and depression are 0.35, a conservative estimate based on previous studies [27],
- the expected intraclass correlation coefficient (ICC estimate .05) based on previous research showing low ICCs within Norwegian schools [28],
- average size of the clusters = 24 (i.e.: number of individuals expected in the EMOTION group within each school in a two-year period).

Because the school sizes were small for some of the more rural areas, it would be impossible to recruit large enough cohorts every semester to run an EMOTION group (minimum of three participants per group). The decision was made to recruit more schools to the study (cluster *N* = 36) to increase the participation in the intervention group. A further consideration driving this decision was that EMOTION groups are limited to a maximum of seven students per group in order to allow for adequate group facilitation by the group leaders. This decision had the knock-on effect of increasing the number of clusters and decreasing within-cluster dependence, which improves the power for detecting smaller effect sizes.

#### Planned statistical analysis

Analysis in the current study will employ regression models controlling for the hierarchical structure of the data to compare the active intervention versus controls adjusting for baseline level. Presentation of the data will be in accordance with Consort guidelines [17]. Several models will be run to test for the main treatment outcomes, implementation outcomes, and related research questions. Missing data will be estimated in the models using full information maximum likelihood estimation, a well-established technique that allows for the inclusion of all available data and estimation of missing values [29].

#### Data monitoring committee

Data quality is monitored by a statistician in the health sciences faculty at UiT, The Arctic University of Norway. This person is responsible for checking that the data are consistent and free from errors and that missing data are accounted for in connection with the Consort guidelines. This person is not one of the investigators in the study, but is employed at one of the participating organizations, RKBU North.

#### Cost

The costs of the interventions will be evaluated by calculating the hours that group leaders and support staff (e.g., supervisors and coordinators) have donated to the project in relation to the number of children treated. An estimate of per-child costs will be included in the final report to funders, along with additional estimates of costs incurred by the trial research team.

## Discussion

The present study is an important contribution to the field regarding working with children with symptoms of anxiety and depression. The results of this study will provide an indication whether or not the EMOTION program is an effective intervention for the prevention of later depression and/or anxiety in children. The study will also provide information about the EMOTION program’s effect on quality of life, self-esteem, and school functioning of the children participating in the study Finally, the project will provide insight into implementation of an indicated intervention for school-aged children within Norwegian health, education, and mental health services.

### National collaboration

The study is an active collaboration project between three regional centers in Norway responsible for work with mental health problems among children and adolescents: RKBU-north, RKBU-mid, and RBUP south and east.

### International collaboration

Professor Philip Kendall at Temple University in Philadelphia, PA, USA and Kevin Stark, University of Texas at Austin, USA, are active participants in the research group and have agreed to host one of the projects doctoral fellows. They have been involved in the project planning, design and are involved in advising, data analysis, and publication of results.

## Trial status

The trial began recruiting in spring, 2014 and is continuing through spring of 2016. Data collection will finish in spring/autumn 2017.

### Trial registration

Clinical Trials NCT02340637, Registered on June 12, 2014, last updated on January 15, 2015.

### Secondary registration

Norwegian Research Council 228846/H10.

### Primary sponsor

RBUP East and South, Gullhaugveien 1-3, 0484 Oslo, mail@r-bup.no.

### Protocol version

April, 2016.

## Ethics and dissemination

### Changes to the protocol

Changes to the project are made in the Standard Operating Procedures (SOP). These changes are recorded and maintained by the principal investigator from RBUP East and South. Changes which are not merely procedural but may impact the experience of the participants in the study are reported to the Regional Committees for Medical and Health Research Ethics for approval.

### Confidentiality

Study participants are provided anonymous study IDs which are store with the collected data. A study key with the participants name and ID are stored in a separate, encrypted file on an internal server at RBUP East and South. Reporting of outcomes will be done using aggregate data to help ensure confidentiality through obscurity.

### Contact for scientific inquiries

Should be addressed to the chief scientific investigator, Simon-Peter Neumer, simon-peter.neumer@r-bup.no.

### Dissemination of results

Will be done through scientific publications, project newsletters, reports to funder(s), and press releases to news media. Three PhD students who are part of the project team will publish and publicly defend dissertations relating to the study. Planned scientific publications include primary outcomes, secondary outcomes, fidelity to the intervention, and implementation. The project team has adopted the Vancouver Protocol for determination of authorship of scientific publications.

## Abbreviations

BPM-P/T: Brief problem monitoring – Parent/Teacher version
BYI-II: Beck Youth Inventory
CAS-CBT: Competence and Adherence Scale for Cognitive Behavioral Therapy
CBCL: Child Behavior Checklist
CBT: Cognitive behavioral therapy
DEFF: Design effect
DMC: Data monitoring committee
ERC: The Emotion Regulation Checklist
HSCL-10: Hopkins Symptoms Checklist
ICC: Intra-class correlation coefficient
KINDL: Children Quality of Life Questionnaire [Kinder Lebensqualität Fragebogen]
MASC: Multidimensional Anxiety Scale for Children
RBUP: Regional Center for Child and Adolescent Mental Health, East and South
RCT: Randomized controlled trial
RKBU: Regional Center for Child and Adolescent Mental Health and Child Welfare
SD: Standard deviation
SMFQ: The Short Mood and Feelings Questionnaire
SOP: Standard operating procedures
TAU: Treatment as usual
